# Generation and Effect Testing of a SARS-CoV-2 RBD-Targeted Polyclonal Therapeutic Antibody Based on a 2-D Airway Organoid Screening System

**DOI:** 10.3389/fimmu.2021.689065

**Published:** 2021-10-18

**Authors:** Yunjiao He, Jing Qu, Lan Wei, Shumin Liao, Nianzhen Zheng, Yingzi Liu, Xingyun Wang, Yue Jing, Clifton Kwang-Fu Shen, Chong Ji, Guxun Luo, Yiyun Zhang, Qi Xiang, Yang Fu, Shuo Li, Yunping Fan, Shisong Fang, Peng Wang, Liang Li

**Affiliations:** ^1^ School of Medicine, Southern University of Science and Technology, Shenzhen, China; ^2^ Institute of Biomedicine and Biotechnology, Shenzhen Institute of Advanced Technology, Chinese Academy of Sciences, Shenzhen, China; ^3^ School of Biomedical Science and Pharmacy, Faculty of Health and Medicine, Hunter Medical Research Institute, University of Newcastle, New Lambton Heights, NSW, Australia; ^4^ Department of Thoracic Surgery, The Seventh Affiliated Hospital of Sun Yat-sen University, Shenzhen, China; ^5^ Department of Otolaryngology, The Seventh Affiliated Hospital of Sun Yat-sen University, Shenzhen, China; ^6^ Department of Otorhinolaryngology Head Neck Surgery, The First Affiliated Hospital of Sun Yat-sen University, Guangzhou, China; ^7^ Guangzhou Key Laboratory of Otorhinolaryngology, Guangzhou, China; ^8^ Department of Research & Development Department, Jiangxi Institute of Biological Products Co. Ltd., Jiangxi, China; ^9^ Department of Research & Development Department, Jiangxi Institute of Biological Products Shenzhen R&D Center Co. Ltd., Shenzhen, China; ^10^ Department of Research & Development Department, Hainan Institute of Pharmaceutical Research Co. Ltd., Hainan, China; ^11^ Department of Otolaryngology, Huazhong University of Science and Technology Union Shenzhen Hospital, Shenzhen, China; ^12^ Department of Otolaryngology, The Sixth Affiliated Hospital of Shenzhen University Health Science Center, Shenzhen, China; ^13^ Department of Pathogen Biology, Shenzhen Center for Disease Control and Prevention, Shenzhen, China

**Keywords:** SARS-CoV-2, viral infection, polyclonal antibody, airway organoids, receptor-binding domain

## Abstract

Coronavirus disease 2019 (COVID-19) is an acute respiratory infectious disease caused by infection with severe acute respiratory syndrome coronavirus 2 (SARS-CoV-2). The US FDA has approved several therapeutics and vaccines worldwide through the emergency use authorization in response to the rapid spread of COVID-19. Nevertheless, the efficacies of these treatments are being challenged by viral escape mutations. There is an urgent need to develop effective treatments protecting against SARS-CoV-2 infection and to establish a stable effect-screening model to test potential drugs. Polyclonal antibodies (pAbs) have an intrinsic advantage in such developments because they can target rapidly mutating viral strains as a result of the complexity of their binding epitopes. In this study, we generated anti–receptor-binding domain (anti-RBD) pAbs from rabbit serum and tested their safety and efficacy in response to SARS-CoV-2 infection both *in vivo* and *ex vivo*. Primary human bronchial epithelial two-dimensional (2-D) organoids were cultured and differentiated to a mature morphology and subsequently employed for SARS-CoV-2 infection and drug screening. The pAbs protected the airway organoids from viral infection and tissue damage. Potential side effects were tested in mouse models for both inhalation and vein injection. The pAbs displayed effective viral neutralization effects without significant side effects. Thus, the use of animal immune serum–derived pAbs might be a potential therapy for protection against SARS-CoV-2 infection, with the strategy developed to produce these pAbs providing new insight into the treatment of respiratory tract infections, especially for infections with viruses undergoing rapid mutation.

## Introduction

Coronavirus disease 2019 (COVID-19), caused by infection with severe acute respiratory syndrome coronavirus 2 (SARS-CoV-2), has threatened global public health for more than a year since it began spreading worldwide at the end of 2019 (1, 2). The complex COVID-19 symptoms range from the asymptomatic to fever, dyspnea, systemic inflammatory syndromes, and pneumonia, as well as death due to multiorgan failure (3, 4). To date, the rapid outbreak of SARS-CoV-2 has resulted in approximately 118 million people infected and more than 2 million deaths (5). Developing efficient therapeutics and vaccines to protect against SARS-CoV-2 infection has become an urgent global priority. Although the World Health Organization (WHO) has claimed the failure of four potential drugs (remdesivir, hydroxychloroquine, lopinavir/ritonavir, and interferon regimens) (6), several promising therapeutics have been developed, with some currently in clinical trials [e.g., immunomodulators and anti-SARS-CoV-2 monoclonal antibodies (mAbs)] (7–10).

SARS-CoV-2 is a member of the beta-coronavirus family, exhibiting 79% nucleotide identity with the sequence of SARS-CoV (1, 11). The SARS-CoV-2 spike (S) protein plays a critical role in mediating viral infectivity, facilitating viral attachment and entry into cells *via* the cell surface receptor angiotensin-converting enzyme 2 (ACE2) (12). Two cleavage sites (S1 and S2) are present in the SARS-CoV-2 S protein (13). The S1 protein contains two independent domains: an N-terminal domain (NTD) and a receptor-binding domain (RBD) (14). Neutralizing the critical domains of the SARS-CoV-2 spike protein is one of the main technical routes employed when using mAbs as COVID-19 therapeutics, as well as in the development of vaccines. In particular, several neutralizing mAbs have been isolated and/or designed to target the RBD (15–17). Although most strategies developed against COVID-19 have obtained emergency use authorization, further characterization of these therapeutics remains necessary (18). There are obvious limitations to using mAbs alone due to viral structural and escape mutations; indeed, more than 2,800 SARS-CoV-2 mutations have been reported to date (19). Mutations appearing in the spike protein have been reported to be more transmissive and extensive (20). The SARS-CoV-2 mutants with mutations B.1.1.7, B.1.351, and P.1 are particularly concerning—especially B.1.1.7, which has been reported in more than 94 countries worldwide (3, 21, 22). A recent study of 19 anti-RBD mAbs has indicated restricted resistance of each mAb in response to viral escape mutants (23). Furthermore, there is much evidence that combinations of mAbs are required to prevent against SARS-CoV-2 (18, 24, 25). Nevertheless, Baum et al. (18) demonstrated that even mAb combinations that overlap the spike protein regions can also fail to neutralize SARS-CoV-2. In addition, with the high cost and lengthy development cycle of a particular single mAb drug, there is growing concern regarding combined mAb-based therapies. Therefore, considering their much lower development costs and shorter bench-to-batch processing, animal-origin polyclonal antibodies (pAbs) are currently reemerging as potential therapeutic candidates for preventing rapid viral mutational escape.

*Escherichia coli* protein expression systems are among the most economical systems for preparing protein antigens. There are nine cysteine residues present in the spike RBD (319–541) sequence, with eight of them connected covalently in the form of disulfide bridges (Cys336–Cys361, Cys379–Cys432, Cys391–Cys525, Cys480–Cys488) (PDB ID: 6LZG) (26). Because disulfide reductase is present in the cytoplasm of *E. coli*, a cysteine-rich RBD protein produced in *E. coli* cannot form its correct disulfide bonds or fold correctly (27). Therefore, for this present study, the RBD protein used as an antigen was overexpressed in an *E. coli* system and subsequently denatured with 6 M guanidine hydrochloride from the inclusion body and purified through a Ni-NTA affinity column. The denatured protein was refolded through fast dilution into refolding buffer containing the glutathione (GSH/GSSG, 5:1) redox couple. The addition of GSH/GSSG provided suitable conditions for reconstitution of the disulfide bonds and enhanced the refolding efficiency of the RBD protein.

In this study, we generated anti-RBD pAbs, effective rabbit pAbs targeting the RBD of the SARS-CoV-2 spike protein, and tested their efficiency and safety in well-differentiated human bronchial epithelial cells (HBECs), as well as *in vivo*. Unlike traditional single-type cell viral infection assays exhibiting no additional host interactions, differentiated HBECs can be used to establish *ex vivo* viral infection models that can help when investigating host responses toward virus infections. Despite still being in its infancy, further drug screening tests and investigations of COVID-19 pathogenesis using differentiated HBECs are both highly feasible and can be performed routinely. Our prospective goal is to generate specific pAbs rapidly, simply, and inexpensively (from rabbits as model species, but ultimately applying horses or other larger species as serum production animals) to defend against many variants of SARS-CoV-2.

## Materials and Methods

### SARS-CoV-2 Spike Receptor-Binding Domain Protein Antigen

The RBD (residues Arg319–Phe541) of the SARS-CoV-2 spike protein was cloned into the pET21a expression vector (Invitrogen) with a C-terminal 6× His tag for purification. The construct was transformed into bacterial BL21 (DE3)-pLysS competent cells, and a single colony was cultured in Luria broth (LB) medium for protein expression. The bacterial pellet was lysed using a high-pressure homogenizer; the target protein was present in inclusion bodies, which were washed with 2 M urea buffer and then solubilized in 8 M urea-containing buffer (50 mM Tris, pH 9.0, 8 M urea, 10 mM beta-ME). The denatured protein was purified through Ni^2+^-affinity chromatography and size exclusion chromatography under denaturing conditions. Protein refolding was performed through fast dilution in refolding buffer (50 mM Tris, pH 9.0, 0.4 M arginine, 5 mM GSH, 0.5 mM GSSG) to decrease the concentration of urea.

### ELISA of Rabbit Serum

A 96-well polystyrene microtiter ELISA (15) plate was coated with recombinant RBD in 1× phosphate buffered saline (PBS) overnight at 4°C and then blocked with 1% bovine serum albumin (BSA) in 1× PBS for 1 h at 37°C. The plates were washed three times with 1× PBST (1× PBS, pH 7.4, with 0.1% Tween-20). Serial dilutions of rabbit sera samples (starting at a 1:1,000 dilution; 100 µl) were added to the coated plate, which was then incubated at 37°C for 1 h. After each step, the plate was washed four times with 1× PBST. Anti-rabbit immunoglobulin G (IgG) horseradish peroxidase (HRP) diluted in 1× PBST (1:1,000) was added (100 µl/well); the plate was incubated for 1 h at 37°C. 3,3´,5,5´-Tetramethylbenzidine (TMB) substrate (100 µl) was added, and the system incubated for 10 min; the reaction was stopped through the addition of 2 N H_2_SO_4_ (50 µl). The absorbances were measured at 450 nm using an ELISA reader (Thermo Fischer Scientific, USA). The ELISA-based determination of the EC_50_ value for ACE2 was measured at OD450. The specificity of the antibodies raised in rabbits was evaluated through a sandwich ELISA using SARS-CoV-2 RBD antigen. Normal rabbit plasma (PBS-immunized) was used as the negative control; anti-RBD antibody purchased from Sino Biological Company was used as the positive control.

### Immunization of Rabbits With Receptor-Binding Domain

Healthy female New Zealand white rabbits (weight: 2.5–3.0 kg) with no detectable antibodies against SARS-CoV-2 were immunized to generate anti-RBD antibodies. Prior to immunization, rabbit sera were collected as negative controls for antibody titer evaluation. Every 2 weeks, the rabbits were immunized intramuscularly with purified RBD proteins mixed with incomplete Freund’s adjuvant (IFA). Rabbit antiserum was obtained 1 week after each injection.

### Cells and Viruses

Vero E6 cells (African green monkey kidney, ATCC CCL-81) were cultured in a 24-well plate in Dulbecco’s Modified Eagle Medium (DMEM; Thermo Fisher Scientific, USA) supplemented with 10% fetal bovine serum (FBS) and penicillin (100 U/ml)–streptomycin (100 μg/ml) at 37°C under 5% CO_2_. SARS-CoV-2 was originally isolated from throat swabs from patients who were positive for SARS-CoV-2 [determined by real-time quantitative polymerase chain reaction (RT-qPCR)]. Virus titers were determined through a standard plaque assay on Vero cells; virus stocks were stored in aliquots at –80°C until required.

### SARS-CoV-2 Pseudovirus Packaging System and Virus Infection

The plasmids PNL4-3 luc R-E (designed to carry the pseudovirus skeleton protein-coded gene and luciferase reporter) and pVAX-Spike (a virus membrane protein-expressing plasmid) were selected using an E.Z.N.A.^®^ Endo-Free Plasmid DNA Maxi Kit (D6926). PNL4-3 luc R-E (8 μg) and pVAX-Spike (4 μg) were co-transfected into 293T cells using Lipo293™ transfection reagent c0521 (Beyotime Biotech, China) to generate the pseudovirus. The 293T-ACE2 cells were seeded in a 96-well plate (8,000 cells/well) overnight, and then the pseudovirus (100 μl) was seeded in DMEM (100 μl) for 1 h to prepare the mixture. Subsequently, the mixture was cocultured with 293T-ACE2 cells for 48 h. The pseudovirus-infected cells were detected using a Luciferase Reporter Gene Assay Kit 11401ES60 (Yeasen Biotech, China). The DMEM was removed, and the cells were washed with PBS. The cell lysate buffer (30 μl) was added to the Eppendorf tube, which was vortexed for 15 min at room temperature and spinned down (10,000 rpm, 15 min). The supernatant was removed to a new Eppendorf tube and mixed with Luciferase Reporter Gene Assay reagents (100 μl). The relative light unit (RLU) of the mixture was detected; the inhibition ratio of the pAbs was calculated by comparing the RLU of the pseudovirus-infected cell lysate with that of the mock control of cell lysate.

### Primary Human Bronchial Epithelial Cell Air–Liquid Interphase Culture

HBECs were obtained from patient biopsy residues of bronchoscopic sampling. Cells were cocultured with mouse fibroblast 3T3. The medium was refreshed every 2 days until confluent. Once the HBECs were confluent in a monolayer, cells were seeded on the apical chamber of a 24-well transwell (Corning transwell 3470) precoated with human collagen I (Advanced Biomatrix, USA). The primary HBECs were cultured in a transwell for differentiation at 37°C under 5% CO_2_. The transwell containing the HBECs was first cultured in a submerged phase. Once the cell layer was intact, the medium on the apical chamber was removed and the cells were subjected to an air–liquid interphase (ALI) culture. The medium was refreshed twice a week, and the HBECs were differentiated for 20 days using PneumaCult ALI media (STEMCELL Technologies, Canada) prior to performing the infection experiment. The studies involving human participants were reviewed and approved by Institution Review Board from Shenzhen Institute of Advanced Technology, Chinese Academy of Sciences, Shenzhen, China (SIAT-IRB-200215-H0414), and Huazhong University of Science and Technology Union Shenzhen Hospital, Shenzhen, China (IRB72656). The patients/participants provided their written informed consent to participate in this study.

### SARS-CoV-2 Infection of Air–Liquid Interface Human Epithelial Cultures

The well-differentiated HBECs were infected with 10,000 plaque-forming units (PFUs) of SARS-CoV-2 per 50 μl in the basal compartment of a transwell plate on day 28 and incubated for 1 h at 37°C. The virus was removed and washed three times with PBS. Each virus-infected transwell was transferred into a new plate (Corning, USA) with serum-free growth medium (05001, 296 Stemcell Technologies, CA, USA; 500 μl) and incubated until harvested at 37°C under 5% CO_2_. At every point in time, PBS (150 μl) was added into the apical compartment of the transwell and harvested for measurement. The samples including the PBS wash and cells were harvested. Total RNA was extracted using TRIzol for RT-qPCR. For immunohistological analysis, the epithelial tissue together with the transwell membrane was collected.

### RNA Sequencing

Total RNA was extracted from the HBECs using TRIzol (Thermo Fisher). The integrity of the total RNA and the level of DNA contamination were assessed using an Agilent 2100 bioanalyzer (Agilent Technologies, Santa Clara, CA, USA). Libraries were constructed using a TruSeq Stranded mRNA LT Sample Prep Kit (Illumina, San Diego, CA, USA). Transcriptome sequencing and analysis were conducted by OE Biotech (Shanghai, China) as described below. The libraries were sequenced on an Illumina Novaseq 6000 platform, and 150-bp paired-end reads were generated.

### Transcriptomic Analysis and Functional Enrichment Analysis

The clean reads were mapped to the human genome (GRCh38) using HISAT2. The fragment per kilobase of exon per million mapped fragments (FPKM) of each gene was calculated using Cufflinks; the read counts of each gene were obtained using HTSeq-count, and differential gene expression was determined using the R statistic package DESeq2 (R/Bioconductor) (28). The following criteria were adopted to filter the unique sequence reads: maximum number of hits for a read, 1; minimum length fraction, 0.9; minimum similarity fraction, 0.8; maximum number of mismatches, 2. A constant of 1 was added to the raw transcript count value to avoid any problems caused by the use of a value of 0. The DESeq2 package was used for statistical analysis of the transcript count table. The transcript counts were normalized to the effective library size. The differentially expressed genes (DEGs) were identified by performing a negative binomial test. Transcripts were determined as differentially expressed when they showed a ≥2-fold change with an adjusted p-value of 0.05. Pathway analysis and data presentation were performed using the software Ingenuity Pathway Analysis (IPA; Qiagen Bioinformatics). Hierarchical cluster analysis of DEGs was performed to demonstrate the expression pattern of genes. Gene Ontology (GO) enrichment and Kyoto Encyclopedia of Genes and Genomes (KEGG) pathway enrichment analysis of DEGs were performed respectively using R based on the hypergeometric distribution. DEGs were imported to the IPA software. Four types of analysis were performed using IPA: canonical pathway analysis, disease and function analysis, graphical summary analysis, and interaction network analysis. The results were filtered by p-value and *Z*-score.

### Anti-Receptor-Binding Domain Polyclonal Antibody Intake *In Vivo* Model

Eight-week-old C57 mice intranasally inhaled 0.2 mg/g (body weight) anti-RBD pAbs or saline control, respectively. Mice in the tail intravenous group were injected separately with 0.2 mg/g (body weight) anti-RBD pAbs or saline control. Lung samples were collected on days 3, 7, and 14 after treatment. H&E staining was performed for histopathologic analysis of the lung tissues. The animal study was reviewed and approved by Institutional Animal Care and Use Committee, Shenzhen Institute of Advanced Technology, Chinese Academy of Sciences (SIAT-IACUC-YYS-LL-A0550).

### Quantification of Viral Gene Expressions by qRT-PCR

Total RNA was extracted from cell lysates using TRIzol. The extracted RNAs were reverse transcribed into cDNA using a PrimeScript RT reagent kit (Takara, Japan). mRNA expression was measured through real-time PCR amplification with the specific kit for ORF1ab/N (BioGerm, China) using an ABI Step One Plus™ Real-Time PCR System (Applied Biosystems, USA).

## Results

**Figure 1:**
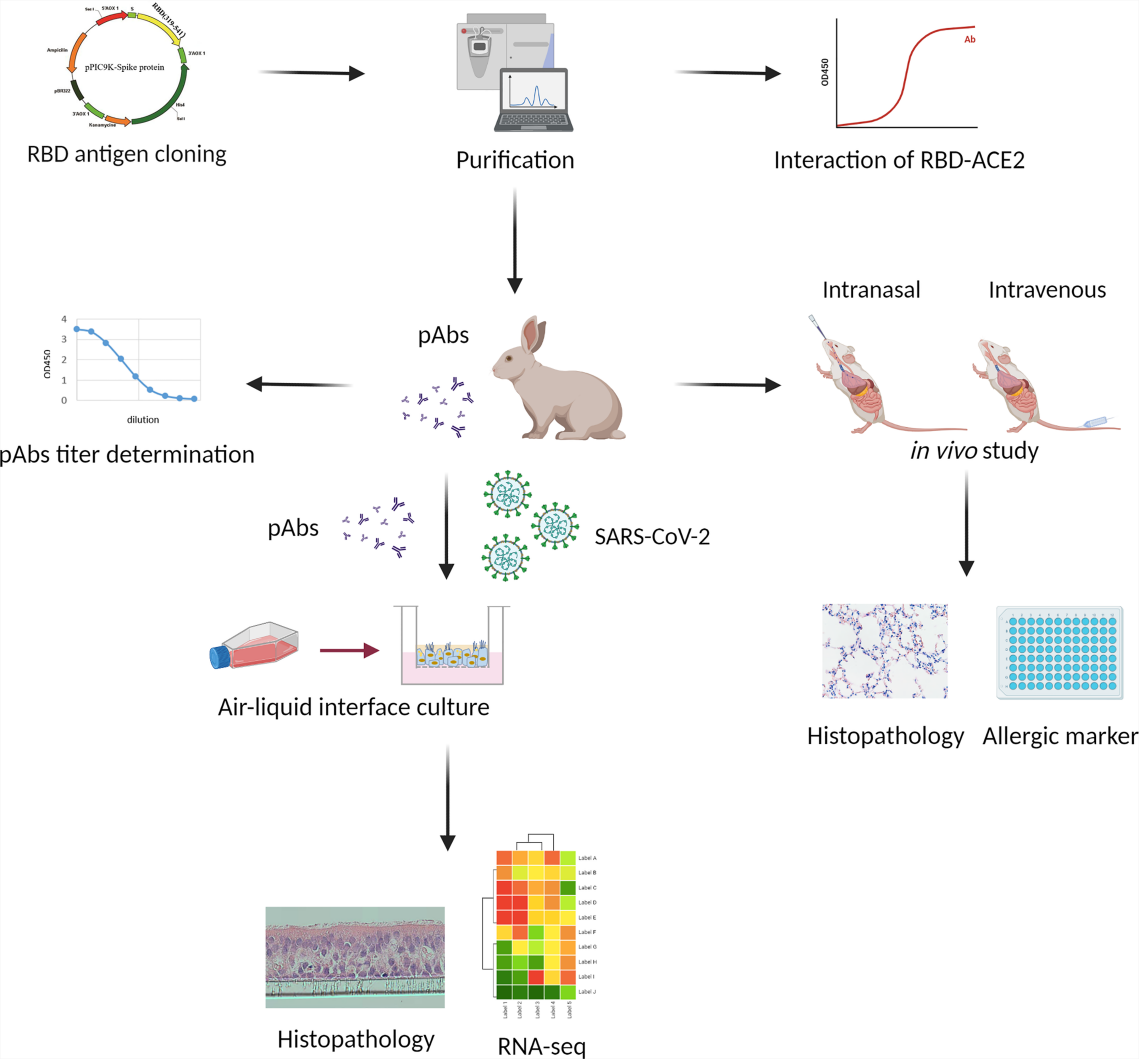
Flowchart of study design. The receptor-binding domain (RBD) antigen was cloned into a vector and purified chromatographically. The anti-RBD polyclonal antibodies (pAbs) extracted from rabbit serum were tested *in vitro* and *in vivo*. The binding efficacy of antibodies was tested in terms of EC_50_. The viral-infected human bronchial epithelial cells (HBECs) cultured in air–liquid interphase (ALI) were used to test the safety and efficacy of the anti-RBD pAbs based on the results of viral quantification, histopathology, and RNA sequencing (RNA-seq) analyses. Finally, the anti-RBD pAbs were tested *in vivo*, and samples were collected for histopathology and allergic marker analysis.

**Figure 1** provides a schematic representation of the approach used in this study. In brief, *E. coli* recombinant expressing SARS-COV-2 spike RBD protein, which displayed high binding ability toward ACE2 protein, was used as the protein antigen to immunize rabbits and generate the rabbit anti-RBD serum. The anti-RBD pAb was purified from the serum through protein A affinity chromatography, and the resulting antibody was sterilized through filtration. Primary HBECs were cultured and progressed into ALI differentiated cultures on transwell inserts. The ALI cultures were maintained until evidence appeared for mature mucus, cilia, and cell differentiation. All of the HBECs were infected with SARS-CoV-2, then treated separately with mock control and anti-RBD pAbs. The airway epithelial morphology was demonstrated through H&E staining. The samples were collected for RNA sequencing (RNA-seq) analysis to detect the gene expression patterns, thereby testing the treatment effects and safety of the antibodies. Our anti-RBD pAbs were also investigated *in vivo* in mouse models through inhalation and tail vein injection. Histopathology analysis and an ELISA for the allergy marker were performed to certify the safety and efficacy of our pAb treatment *in vivo*.

### Preparation of Receptor-Binding Domain Antigen and Production of Rabbit Polyclonal Antibodies

**Figure 2:**
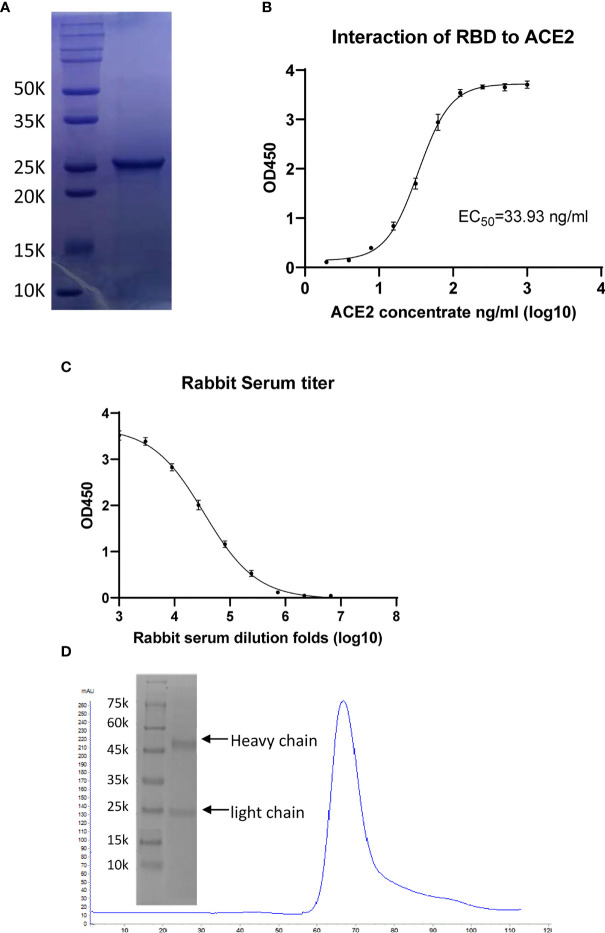
Characterization of receptor-binding domain (RBD) protein antigen and the generated polyclonal antibodies (pAbs). **(A)** Sodium dodecyl sulfate–polyacrylamide gel electrophoresis (SDS-PAGE) of purified RBD protein having a molecular weight (MW) of approximately 26 kDa. **(B)** ELISA of the binding of angiotensin-converting enzyme 2 (ACE2) to immobilized RBD, giving an EC_50_ value of 33.93 ng/ml. **(C)** ELISAs checking the antibody titers of rabbit serum before and after immunization. **(D)** Purification of anti-RBD pAbs through protein-A affinity and detection through SDS-PAGE.

The RBD gene was subcloned into pET-21a (+) vector and expressed by transformation in BL21 (DE3) pLysS. The protein was purified through size-exclusion chromatography, following standard procedures. The target protein was eluted out in a single sharp peak with a molecular weight of approximately 26 kDa, detected using sodium dodecyl sulfate–polyacrylamide gel electrophoresis (SDS-PAGE) (**Figure 2A**). The obtained protein was sterilized through filtration for later immunization. Because the binding of RBD to the ACE2 receptor on host cells is critical for viral infection, we used a functional ELISA to detect the binding of RBD to hACE2 protein. The immobilized RBD at 2 μg/ml (100 μl/well) could bind human ACE2 protein; the EC_50_ value of ACE2 was 33.93 ng/ml (**Figure 2B**).

For preparation of the RBD protein antiserum, rabbits were immunized five times with RBD protein with a 2-week interval. The rabbit sera prior to immunization were collected as negative controls for antibody evaluation. Every 2 weeks, the rabbits were immunized with RBD protein mixed with IFA, and the antisera were collected 1 week after each injection. The titer of serum was evaluated through an indirect ELISA (15). In brief, polystyrene microwell plates were coated with RBD protein antigen (100 µl/well containing 6.0 µg/ml RBD protein) and washed with PBST; serially 3-fold-diluted serum samples (from 1:1,000 initially to 1:6,561,000 finally) were added to the plates and incubated at 37°C for 1 h. HRP-conjugated goat anti-rabbit IgG diluted 1,000-fold and the substrate TMB solution were used for detection. An absorbance at 450 nm greater than twice those of the negative controls was considered positive; the antiserum titer was defined as the highest dilution that still gave a positive value. The antiserum gave a high signal after 1/1,000 dilution, with the signal gradually declining with subsequent dilutions and reaching the cutoff value at 1/2,187,000 dilution. Collectively, the titer of anti-serum was 2,187,000 (**Figure 2C**). The corresponding signal of the negative control was very low, indicating that the serum prior to immunization did not react with RBD (**Figure 2D**). Antibody purification was performed using a 5-ml protein-A affinity column; the pAbs were eluted out using 0.1 M glycine (pH 3.0) buffer and dialyzed into PBS buffer for the neutralization assay (**Figure 2D**).

### Construction of Well-Differentiated Human Bronchial Epithelial Cells and SARS-CoV-2 Infection

**Figure 3:**
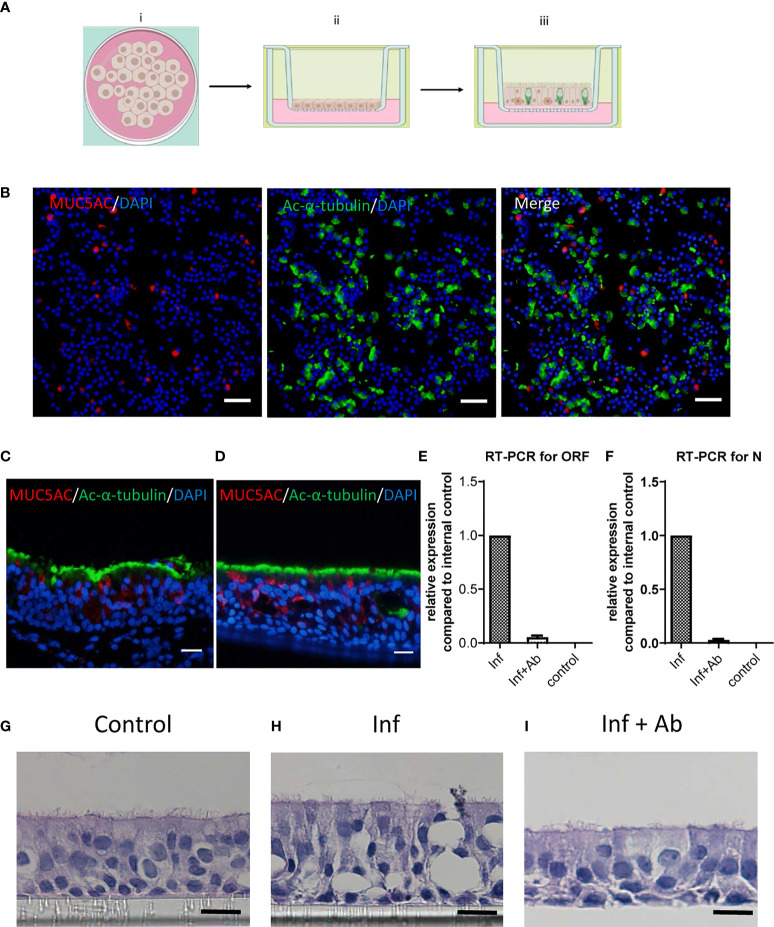
Infection on differentiated human bronchial epithelial cells (HBECs). **(A)** Schematic representation of air–liquid interphase (ALI) culture conditions and differentiation. **(B)** Immunofluorescence staining of differentiated HBECs revealing Ac-α-tubulin stained in ciliated cells (green), goblet cells stained with MUC5AC (red), and the 4',6-diamidino-2-phenylindole (DAPI)-represented nucleus (blue). Scale bar: 50 μm. **(C, D)** Immunofluorescence staining of **(C)** bronchial biopsy and **(D)** cultured HBECs with ciliated cells stained with Ac-α-tubulin (green) and goblet cells stained with MUC5AC (red). Scale bar: 20 μm. **(E, F)** RT-qPCR quantification of **(E)** ORF and **(F)** N domains of severe acute respiratory syndrome coronavirus 2 (SARS-CoV-2) in topical secretions. **(G–I)** H&E images revealing **(G)** the tissue integrity of the mock-infected control (Control), **(H)** tissue damage following SARS-CoV-2 infection (Inf), and **(I)** the protected epithelium with anti-receptor-binding domain (RBD) polyclonal antibody (pAb) treatment after SARS-CoV-2 infection (Inf + Ab). Scale bar: 10 μm.

The primary HBECs were seeded in transwell inserts and cultured in ALI for 20 days to become well-differentiated, forming 2-D airway organoids (**Figure 3A**). Cells exposed to air differentiated into a pseudostratified epithelium with goblet cells and ciliated cells and produced mucus. Immunofluorescence staining revealed the epithelial cell differentiation (**Figure 3B**). The goblet cells were stained red for MUC5AC; the ciliated cells were stained green for Ac-α-tubulin. The images indicated that the HBECs were well-differentiated in the transwell. Moreover, similar staining patterns of bronchial biopsy (**Figure 3C**) and cultured bronchial epithelial cells (**Figure 3D**) with these cell markers indicated that the airway organoid system could mimic the structure of the human bronchial epithelium, suggesting the feasibility of using airway organoids as the effect testing system for the antibodies. The topical secretion of SARS-CoV-2 was measured using a standard SARS-CoV-2 RT-qPCR kit. The contents of both the ORF1ab (**Figure 3E**) and N (**Figure 3F**) RNA of the virus decreased upon treatment with the anti-RBD pAbs (Inf+Ab) compared with the infected group (Inf). The control group represents the cells without viral infection, resulting in no detectable viral gene. Interestingly, the anti-RBD pAbs contributed to protect the HBECs from tissue damage caused by SARS-CoV-2 infection. The HBECs were well-differentiated in the ALI cultures and formed tight epithelium without viral infection (**Figure 3G**). After SARS-CoV-2 infection, severe damage appeared in the tissue structure of the airway organoids (**Figure 3H**). Interestingly, there were no significant differences between the anti-RBD pAb–treated HBECs (**Figure 3I**) and the control group (**Figure 3G**). The results of the RT-qPCR analysis of SARS-CoV-2, and the H&E staining images, revealed the ability of the anti-RBD pAbs to inhibit viral replication and protect the integrity of the epithelial tissue of the HBECs from viral infection.

### Neutralizing Potential of Polyclonal Antibodies Against Mutations of SARS-CoV-2

**Figure 4:**
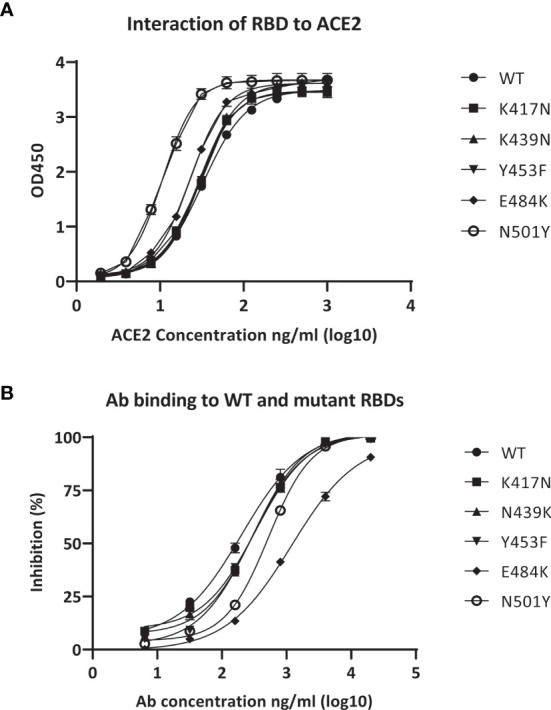
Binding activity of anti-receptor-binding domain (RBD) polyclonal antibodies (pAbs) with various severe acute respiratory syndrome coronavirus 2 (SARS-CoV-2) mutant RBDs. **(A)** Binding of angiotensin-converting enzyme 2 (ACE2) to immobilized RBDs from wild-type (WT) SARS-CoV-2 and mutants. **(B)** Binding of anti-RBD pAbs with WT and mutant SARS-CoV-2 RBDs.

We assessed the neutralizing activity of the pAbs against several widely spread SARS-CoV-2 mutants, including K417N, K439N, Y453F, E484K, and N501Y, by measuring the binding abilities of the pAbs to the mutant RBDs. Measurements of the binding abilities of RBD wild-type (WT) and other SARS-CoV-2 mutant RBDs toward ACE2, determined through a functional ELISA, revealed that the SARS-CoV-2 WT and mutant RBDs could be recognized by the ACE2 receptor, enabling potential infection of the cells (**Figure 4A**). In addition, we observed the binding ability of the pAbs toward the RBD of WT SARS-CoV-2 and mutants (**Figure 4B**). The EC_50_ values of pAbs toward SARS-CoV-2 WT and the K417N, K439N, Y453F, E484K, and N501Y mutant RBDs were 201.9, 330.4, 309.3, 280.9, 1198, and 524.3 ng/ml, respectively, suggesting efficient neutralization potential of our pAbs toward the tested various SARS-CoV-2 mutants.

The EC_50_ value of the E484K mutant was, however, 5-fold higher than that of the WT. The relatively lower performance against the E484K mutant piqued our interest; accordingly, we employed the E484K mutant pseudovirus to perform a neutralization assay using the SARS-CoV-2 pseudovirus packaging system to serve as a closer mimic of the viral infection and to confirm the suitability of our polyclonal therapy in the treatment of E484K-related variants. The data revealed that the inhibition ratio of our pAbs against the E484K mutant was generally similar to that for the WT SARS-CoV-2 (**Supplementary Figure 2**), indicating that the pAbs could have good neutralizing activity toward both the WT SARS-CoV-2 and the E484K mutant.

### Anti-Receptor-Binding Domain Polyclonal Antibody Inhalation and Tail Intravenous Injection *In Vivo* Study

**Figure 5:**
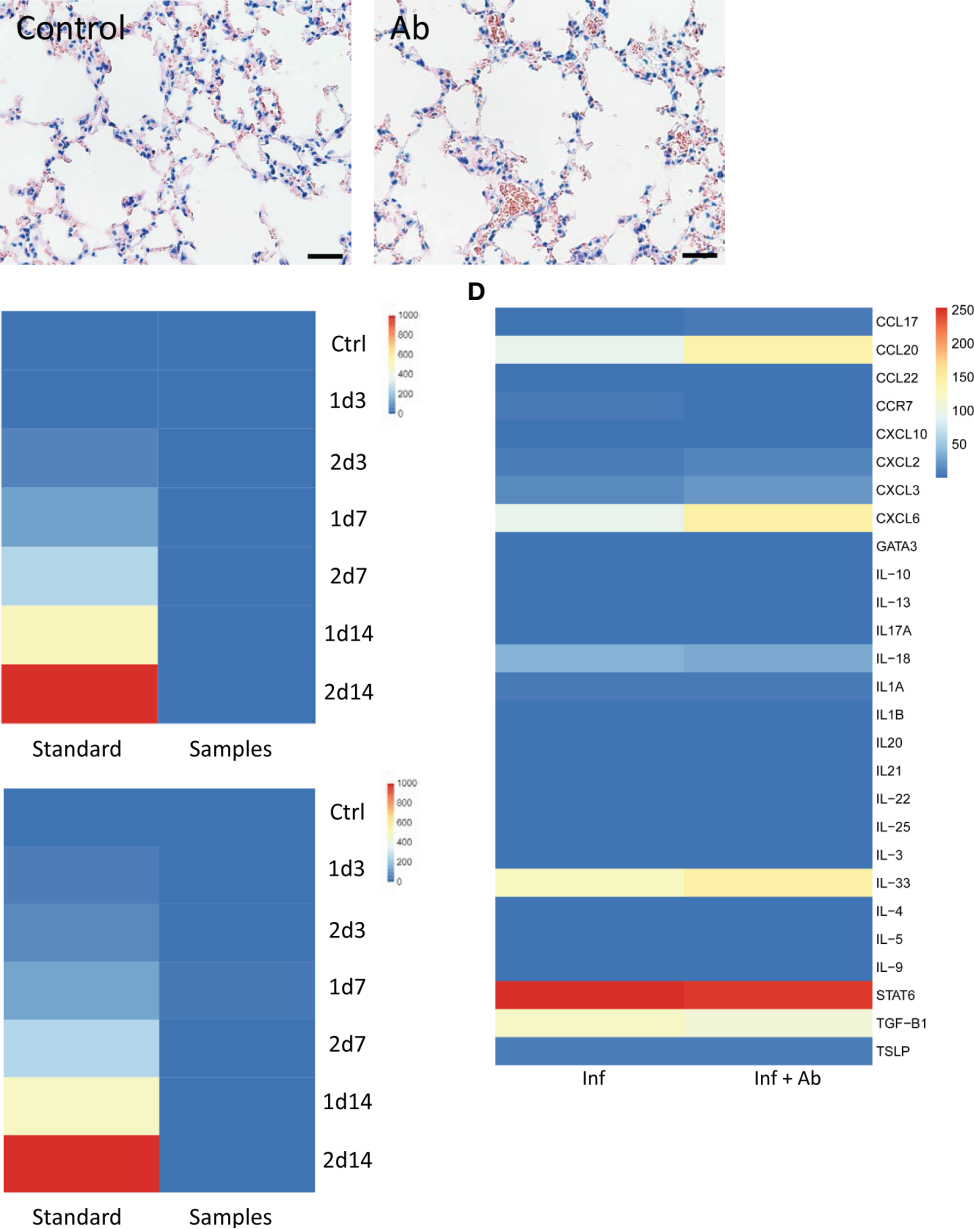
Immune effects of the antibody. **(A)** H&E-stained lung sections from mouse that inhaled saline *vs*. anti-receptor-binding domain (RBD) polyclonal antibodies (pAbs). Scale bar: 80 μm. **(B, C)** Negligible secretion of major allergy-related cytokine interleukin (IL)-13 detected in the presence of pAbs *in vivo* in the **(B)** inhalation intake model and **(C)** tail intravenous injection model. **(D)** Heatmap of immune markers from organoid RNA sequencing (RNA-seq) analysis.

The airway epithelium is usually the first line of defense against respiratory viral infection (29). In general, inhalation therapies targeting the airway epithelium can thus be used in the case of early infection, while intravenous injection can be efficient against severe infection. Therefore, in this study, we designed two methods to mimic the comprehensive situation for infectious diseases. We applied mouse inhalation and tail intravenous injection of anti-RBD pAbs in mouse models to investigate the potential immune side effects of our antibodies. H&E staining of lung sections at day 3 posttreatment indicated no significant immune cell infiltration from either the saline control or anti-RBD pAb treatment groups (**Figure 5A**). In addition, we collected the serum to measure the secretion of interleukin (IL)-13, one of the critical cytokines contributing to allergic responses, after the inhalation and tail intravenous anti-RBD pAb treatments. The concentrations of IL-13 in all of the samples were below the minimum detection range of the ELISA kit. The ELISA results presented as heatmaps indicate that the anti-RBD pAbs had no significant influence on the secretion of the allergy-relevant cytokine IL-13 through either intranasal or tail intravenous intake *in vivo* (**Figures 5B, C** **)**, demonstrating that the anti-RBD pAbs did not induce significant allergic responses in the mouse models.

Based on RNA-seq analysis, we found that chemokine ligand 20 (CCL20), chemokine (C-X-C motif) ligand 8 (CXCL8), interleukin 33 (IL-33), signal transducer and activator of transcription 6 (STAT6), and transforming growth factor beta 1 (TGF-β1) were upregulated in both the presence and absence of the anti-RBD pAbs in the infected HBECs (**Figure 5D**). Several key immune markers have been identified in clinical studies of COVID-19, including IL-8, TGF-β1, IL-33, CXCL8, and CXCL10 (30–33). The chemokine CXCL8 has been induced by viral infection, contributing to lung injury (31). The similar levels of expression of the allergic markers in our case indicate, however, that the addition of anti-RBD pAb treatment did not induce more severe host responses in the infected HBECs. The analyses of allergic markers from both *in vivo* and *ex vivo* studies suggested the safety of our anti-RBD pAbs.

### Transcriptomic Profile in the Presence of Anti-Receptor-Binding Domain Polyclonal Antibodies in Human Bronchial Epithelial Cells Following SARS-CoV-2 Infection

The DEGs from the SARS-CoV-2–infected HBECs with addition of control IgG (with respect to the non-infected HBEC group) and from the anti-RBD pAb treatment group (with respect to the IgG control in HBECs following SARS-CoV-2 infection group) were imported into the IPA software. The cellular signaling pathway histogram presents the most relevant canonical pathways ranked in terms of –log(*p*-value). The top enriched relevant diseases and functions were analyzed with both upregulated and downregulated genes (p < 0.05) using the IPA software (**Supplementary Figure 3A**). The immunological disease–, respiratory disease–, and infectious disease–associated functions were significantly modulated in the SARS-CoV-2–infected HBECs with control IgG treatment relative to the non-infected HBECs, confirming successful infection when using our well-differentiated airway epithelial infection model. Next, we identified the statistically enriched terms, including canonical pathways (**Supplementary Figure 3B**) and GO terms (**Supplementary Figure 4A**). The interferon (IFN) signaling pathway and mitogen-activated protein kinase (MAPK) signaling pathway were present on both the summary graph and enriched ontology cluster bar chart, indicating the induction of antiviral host responses in the HBECs after SARS-CoV-2 infection. More specifically, we selected a subset of the representative term “virus entry related function” from this cluster and converted it into a network layout (**Supplementary Figure 4B**). Several viral entry–associated genes were upregulated in the SARS-CoV-2–infected HBECs, including chimeric antigen receptor (CAR), filamentous actin (F-actin), integrin alpha-5 (Integrin α5), integrin beta (Integrin β), Ras, and protein kinase C (PKC).

We used the same strategy to analyze the transcriptomic profiles of the SARS-CoV-2–infected HBECs combined with anti-RBD pAb treatment relative to the IgG control in HBECs following SARS-CoV-2 infection group. The top enriched relevant diseases and functions were plotted in **Supplementary Figure 5A**. The top enriched pathways were represented by a network (**Supplementary Figure 5B**); the enriched ontology cluster was used to reveal the GO terms (**Supplementary Figure 6A**). Interestingly, the positive regulations of epithelial cell physiological activities were evident in the top GO classification, supporting our results from the *ex vivo* study using the well-differentiated HBECs, suggesting a critical role of anti-RBD pAb treatment in tissue protection. We selected a specific subset of the representative term “airway pathology” from the canonical pathways (**Supplementary Figure 6B**). The DEGs related to airway pathology in the SARS-CoV-2–infected HBECs treated with the anti-RBD pAbs are highlighted in the graph (e.g., CXCL1, CCL20, IL-8).

## Discussion

Although antibody therapeutics against the SARS-CoV-2 spike protein are promising for fighting the COVID-19 pandemic, there are serious concerns about upcoming mutations leading to drug resistance. Indeed, viral escape mutants present serious challenges for mAb-based therapeutics and, at the same time, they decrease the protection efficiency of current vaccines. Recent findings have been alarming—especially reports that the Novavax and Johnson & Johnson vaccines did not display their expected efficacies in South Africa (34). pAb-based therapeutics are a promising prospect for overcoming such challenges due to their intrinsic variance in antigen binding. A previous study mimicking various drug administration routes (including intranasal administration and nasal turbinate) for the introduction of neutralizing antibodies against SARS-CoV-2 infection in hamsters found that application *via* nasal turbinate was a most effective means of antibody intake (35). Consequently, applying pAb-based therapeutics directly inside nasal turbinates could be feasible and practical prophylaxis to protect against SARS-CoV-2 infection.

In this paper, we report that anti-RBD pAbs significantly decrease SARS-CoV-2 viral RNA copies in infected primary HBECs. ALI cultures of HBECs allowed the direct study of a differentiated pseudostratified epithelium composed of all the cells naturally present in the bronchial epithelium. RT-qPCR analyses and H&E staining of the ALI culture sections after SARS-CoV-2 infection in the presence of anti-RBD pAbs reached the same conclusion: significant protection from SARS-CoV-2 in the HBECs. The RNA-seq results determined *ex vivo* and an ELISA of IL-13 determined *in vivo* also certified that these anti-RBD pAbs did not induce allergic responses. Our *in vivo* study found no significant differences in lung tissue morphology or pathology between a saline control and antibody treatment. Our findings suggest the safety and efficiency of anti-RBD pAbs against SARS-CoV-2 infection.

A previous study in Argentina found that an equine-based pAb therapy was safe and effective against COVID-19 (36). That antibody, designed to target the RBD of the viral S protein, was approximately 50 times more efficient than normal convalescent plasma. A subsequent drug product, INM005 (CoviFab^®^), has been approved to treat COVID-19 patients in Argentina. Moreover, another study has illustrated that the neutralization ability of antibodies generated from horses immunized with SARS-CoV-2 infection was 80 times higher than that of human convalescent plasma (37). Recent reports have demonstrated that immunized animal–derived antibodies have high neutralizing activity, product consistency, and polyvalency (37, 38). One of the major limitations of using rabbit serum is that it is possible to collect only a very small amount of serum from each animal. For the next step of our therapy development following the validation of the anti-RBD pAb development strategy presented here, we should be able to obtain large amounts of pAbs from immunized horses as a more practical product routine. According to our previous equine antibody therapy development experience, this strategy should be rapid, efficient, and cost-effective, producing a large amount of neutralizing antibodies because much more serum can be collected from a single horse, leading to a much higher output of pAbs.

During the COVID-19 pandemic era, there continue to be great concerns about reinfection, as other studies have suggested (21). We are not able to predict how the pandemic will proceed due to the rapidly increasing number of viral variants. Our proposed approach for the development of a pAb therapy has generated a relatively robust system for treatment with efficacy against various mutants of the spike protein of SARS-CoV-2, demonstrating antibody neutralization toward RBD mutant proteins and the RBD mutant pseudovirus of SARS-CoV-2 (**Figure 4** and **Supplementary Figure 2**). This is particularly important because the immune protection provided at present by vaccination is seriously challenged by the spread of the Delta strain of SARS-CoV-2. Our proposed routine for applying this cost-effective antibody therapy with robust performance against mutant spike proteins is through inhalation by the patients in the early stages of infection or through combined administration involving both inhalation and injection for severe cases. While the Delta strain infects and spreads among the vaccinated population (39), viral infection and replication in the airway epithelium will presumably be key factors requiring intervention. We have observed neutralization when applying the pAbs on airway organoids, leading to significant decreases in viral infection and replication in the airway epithelium (**Figure 3**). **Supplementary Figure 4** reveals that the SARS-CoV-2–infected airway epithelium underwent upregulation of genes associated with pro-inflammatory (e.g., IL-6 and IL-17) and antiviral (e.g., IFNs and PKC) responses. Antibody neutralization protected the airway epithelium from extensive infection and tissue damage and reduced the upregulation of inflammatory responses, suggesting that such antibody treatment could protect the epithelium from viral infection and an inflammatory cytokine storm (**Figure 3** and **Supplementary Figure 5**).

With the spread of variants, such as the Delta strain, SARS-CoV-2 is heading in a direction in which it might eventually escape from our current treatment regimens and other preventive measures. If the SARS-CoV-2 virus continues to spread wildly and evolve within infected hosts to produce more crucial mutations, we will be forced to continue monitoring its changes, just as we do currently for influenza viruses. For such information to be useful and practical, we must have the ability to develop new and affordable therapeutics as soon as we can identify new pathogens and provide therapies with robust effects against mutation. The strategy we describe in this paper provides a basic approach for the design and generation of pAbs that could potentially be used as a potential means of passive immune protection against current SARS-CoV-2 strains and emerging new viral mutants, ideally helping us respond rapidly to any new outbreaks.

## Data Availability Statement

The data presented in the study is deposited in the NCBI’s the Sequence Read Archive database, accession number PRJNA762161.

## Ethics Statement

The studies involving human participants were reviewed and approved by Institution Review Board from Shenzhen Institute of Advanced Technology, Chinese Academy of Sciences, Shenzhen, China (SIAT-IRB-200215-H0414), and Huazhong University of Science and Technology Union Shenzhen Hospital, Shenzhen, China (IRB72656). The patients/participants provided their written informed consent to participate in this study. The animal study was reviewed and approved by Institutional Animal Care and Use Committee, Shenzhen Institute of Advanced Technology, Chinese Academy of Sciences (SIAT-IACUC-YYS-LL-A0550).

## Author Contributions

LL, PW, SF, YH, JQ, and YJ contributed to the study design. YH, JQ, LW, SF, SML, NZ, YL, XW, CK-FS, CJ, YZ, QX, YF, GL, SL, YPF, SF, and LL contributed to the experiments. YH, JQ, LW, CK-FS, and LL contributed to the data analysis. LW, LL, YH, PW, and JQ contributed to the article. All authors contributed to the article and approved the submitted version.

## Funding

The study was supported by Shenzhen Science and Technology Innovation Commission for Research and Development Project (JSGG20200207161928126), the National Natural Science Foundation of China (81900071), the Natural Science Foundation of Guangdong Province of China (2021A1515010004), Shenzhen Science and Technology Program (JCYJ20210324115611032 and KQTD20200909113758004) and COVID-19 Prevention and Control Special Project of Guangdong Education Department (2020KZDZX1181). Liang Li was supported by the Research Initiation Fund for Introduction of Talents from Shenzhen Institute of Advanced Technology, Chinese Academy of Sciences (Y9G077).

## Conflict of Interest

YJ, CK-FS, CJ, and GL are currently employed by Jiangxi Institute of Biological Products Co. Ltd., Jiangxi, China, and Jiangxi Institute of Biological Products Shenzhen R&D Center Co. Ltd., Shenzhen, China. YZ is employed by Hainan Institute of Pharmaceutical Research Co. Ltd., Hainan, China.

The remaining authors declare that the research was conducted in the absence of any commercial or financial relationships that could be construed as a potential conflict of interest.

## Publisher’s Note

All claims expressed in this article are solely those of the authors and do not necessarily represent those of their affiliated organizations, or those of the publisher, the editors and the reviewers. Any product that may be evaluated in this article, or claim that may be made by its manufacturer, is not guaranteed or endorsed by the publisher.

## Supplementary Material

Supplementary Figure 1 Protein sequence of SARS-CoV-2 Spike (GenBank no. MN908947) RBD. MRVQPTESIVRFPNITNLCPFGEVFNATRFASVYAWNRKRISNCVADYSVLYNSASFSTFKCYGVSPTKLNDLCFTNVYADSFVIRGDEVRQIAPGQTGKIADYNYKLPDDFTGCVIAWNSNNLDSKVGGNYNYLYRLFRKSNLKPFERDISTEIYQAGSTPCNGVEGFNCYFPLQSYGFQPTNGVGYQPYRVVVLSFELLHAPATVCGPKKSTNLVKNKCVNFHHHHHH. M is added as the starting amino acid, and HHHHHHH is added as the tag for purification.

Supplementary Figure 2 Neutralizing activity of anti-RBD pAbs against WT *vs*. E484K mutant SARS-CoV-2 strains using pseudovirus. Inhibition ratios of pAbs against WT and the E484K mutant SARS-CoV-2 pseudovirus were shown to be similar.

Supplementary Figure 3 Host responses in HBECs following SARS-CoV-2 infection, compared with the control. **(A)** Top 20 enriched relevant diseases and functions modulated in HBECs in response to SARS-CoV-2 infection (*p*-value < 0.05). **(B)** Summary of enriched signaling pathways following SARS-CoV-2 infection.

Supplementary Figure 4 Gene ontology and pathway patterns for host responses in HBECs following SARS-CoV-2 infection, compared with the control. **(A)** Top enriched ontology clusters following viral infection. **(B)** Viral entry related differentially expressed genes modulated in SARS-CoV-2 infected HBECs, compared with non-infected HBECs.

Supplementary Figure 5 Host responses in SARS-CoV-2–infected HBECs in the presence and absence of anti-RBD pAbs. **(A)** Top 20 enriched relevant diseases and functions modulated in HBECs in response to SARS-CoV-2 infection in the presence and absence of anti-RBD pAbs (*p*-value < 0.05); –log(*P*-values) are plotted on the *x*-axis. **(B)** Summary of enriched pathways following SARS-CoV-2 infection with or without anti-RBD pAb treatment.

Supplementary Figure 6 Gene ontology and pathway patterns for host responses in SARS-CoV-2–infected HBECs in the presence and absence of anti-RBD pAbs. **(A)** Top enriched ontology clusters following SARS-CoV-2 infection in the presence and absence of anti-RBD pAbs. **(B)** Airway pathology–related differentially expressed genes modulated in SARS-CoV-2–infected HBECs combined with anti-RBD pAbs treatment.
