# Supplementary figures and images for: Generation and Effect Testing of a SARS-CoV-2 RBD-Targeted Polyclonal Therapeutic Antibody Based on a 2-D Airway Organoid Screening System
Source: Front Immunol. 2021 Oct 18;12:689065. doi: 10.3389/fimmu.2021.689065 (PMC8559598; doi:10.3389/fimmu.2021.689065)

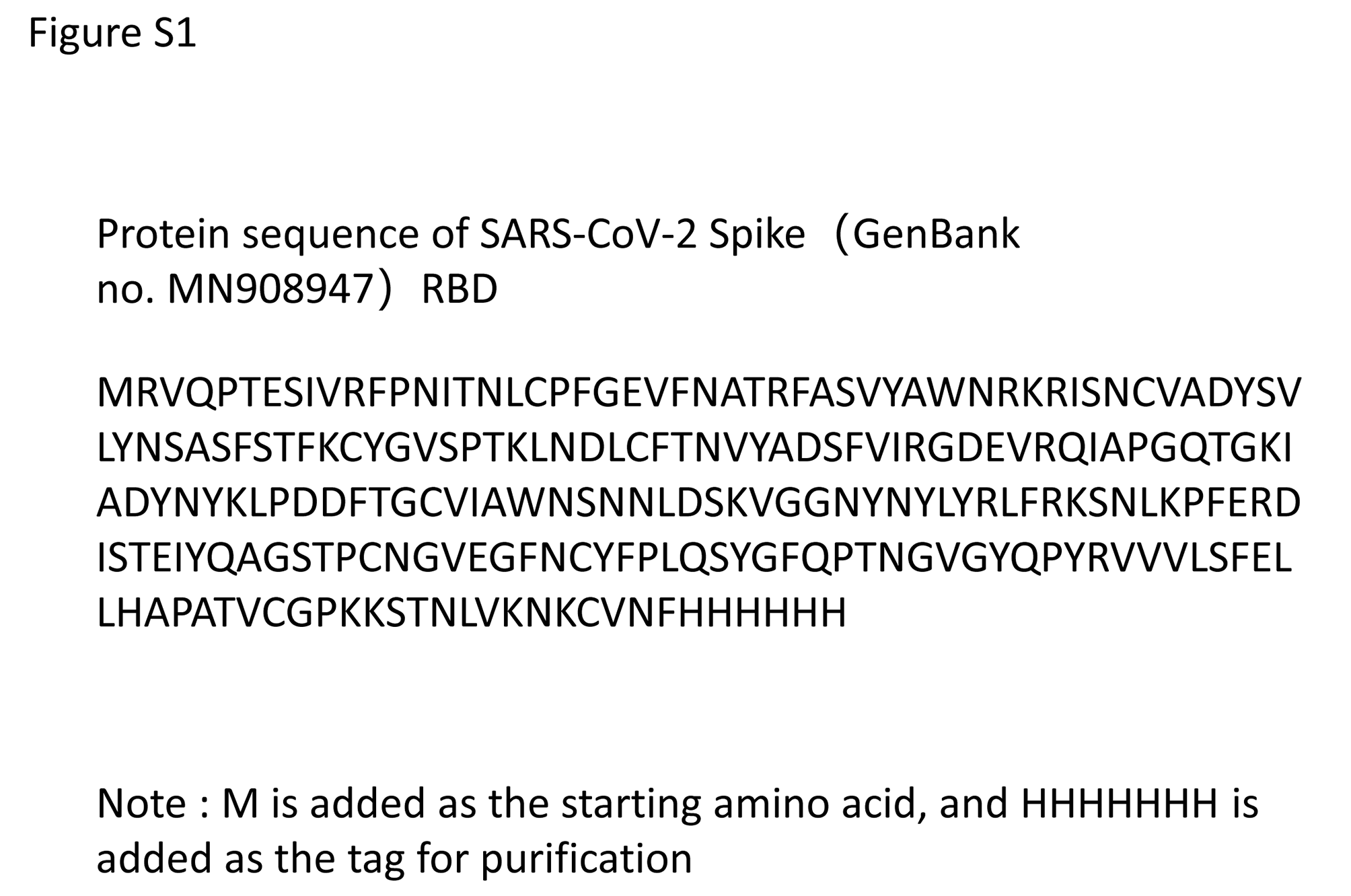

Supplement: Supplementary Figure 1 — Protein sequence of SARS-CoV-2 Spike (GenBank no. MN908947) RBD. MRVQPTESIVRFPNITNLCPFGEVFNATRFASVYAWNRKRISNCVADYSVLYNSASFSTFKCYGVSPTKLNDLCFTNVYADSFVIRGDEVRQIAPGQTGKIADYNYKLPDDFTGCVIAWNSNNLDSKVGGNYNYLYRLFRKSNLKPFERDISTEIYQAGSTPCNGVEGFNCYFPLQSYGFQPTNGVGYQPYRVVVLSFELLHAPATVCGPKKSTNLVKNKCVNFHHHHHH. M is added as the starting amino acid, and HHHHHHH is added as the tag for purification. [file Image_1.tif]

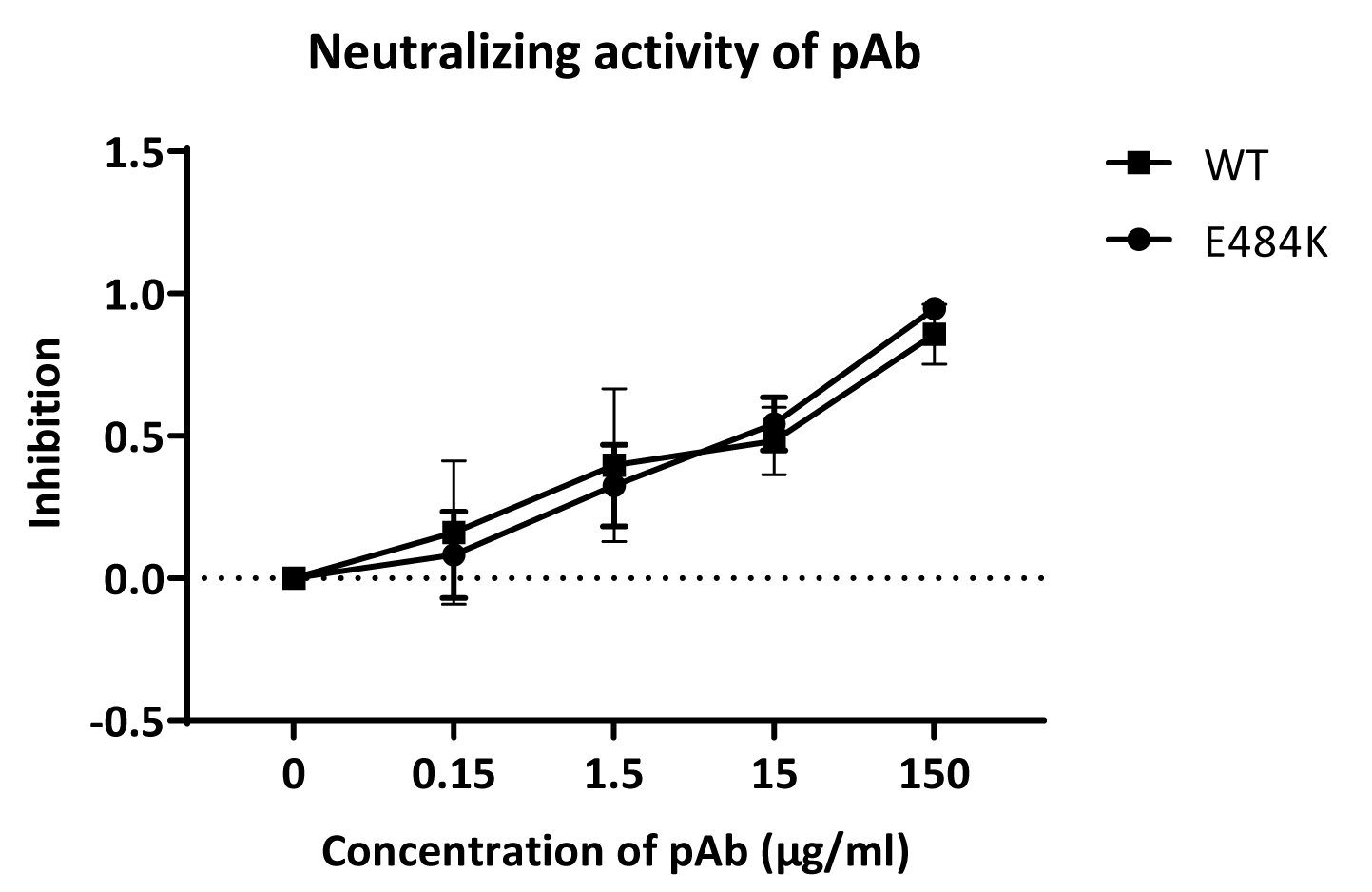

Supplement: Supplementary Figure 2 — Neutralizing activity of anti-RBD pAbs against WT vs. E484K mutant SARS-CoV-2 strains using pseudovirus. Inhibition ratios of pAbs against WT and the E484K mutant SARS-CoV-2 pseudovirus were shown to be similar. [file Image_2.tif]

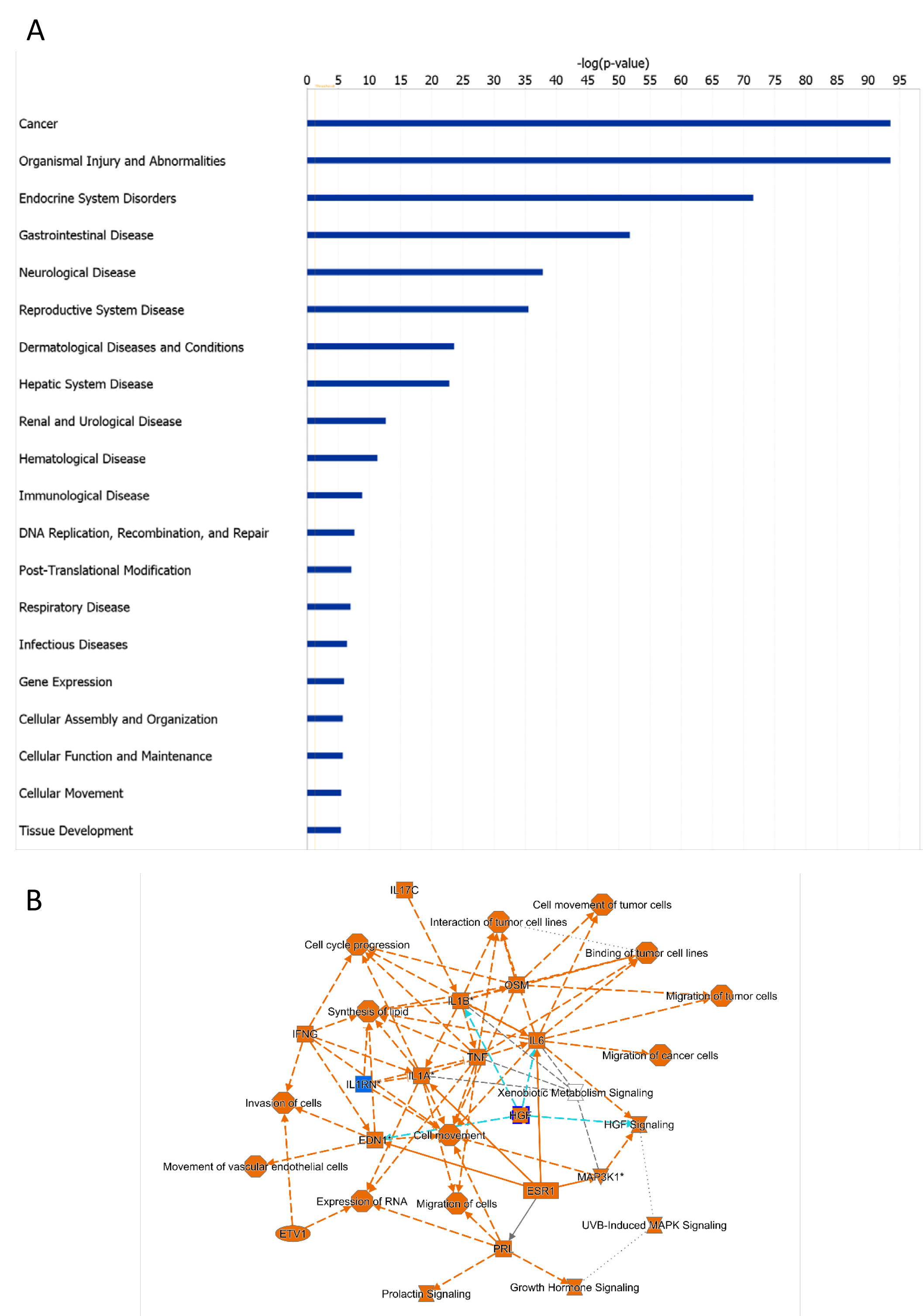

Supplement: Supplementary Figure 3 — Host responses in HBECs following SARS-CoV-2 infection, compared with the control. (A) Top 20 enriched relevant diseases and functions modulated in HBECs in response to SARS-CoV-2 infection (p-value < 0.05). (B) Summary of enriched signaling pathways following SARS-CoV-2 infection. [file Image_3.tif]

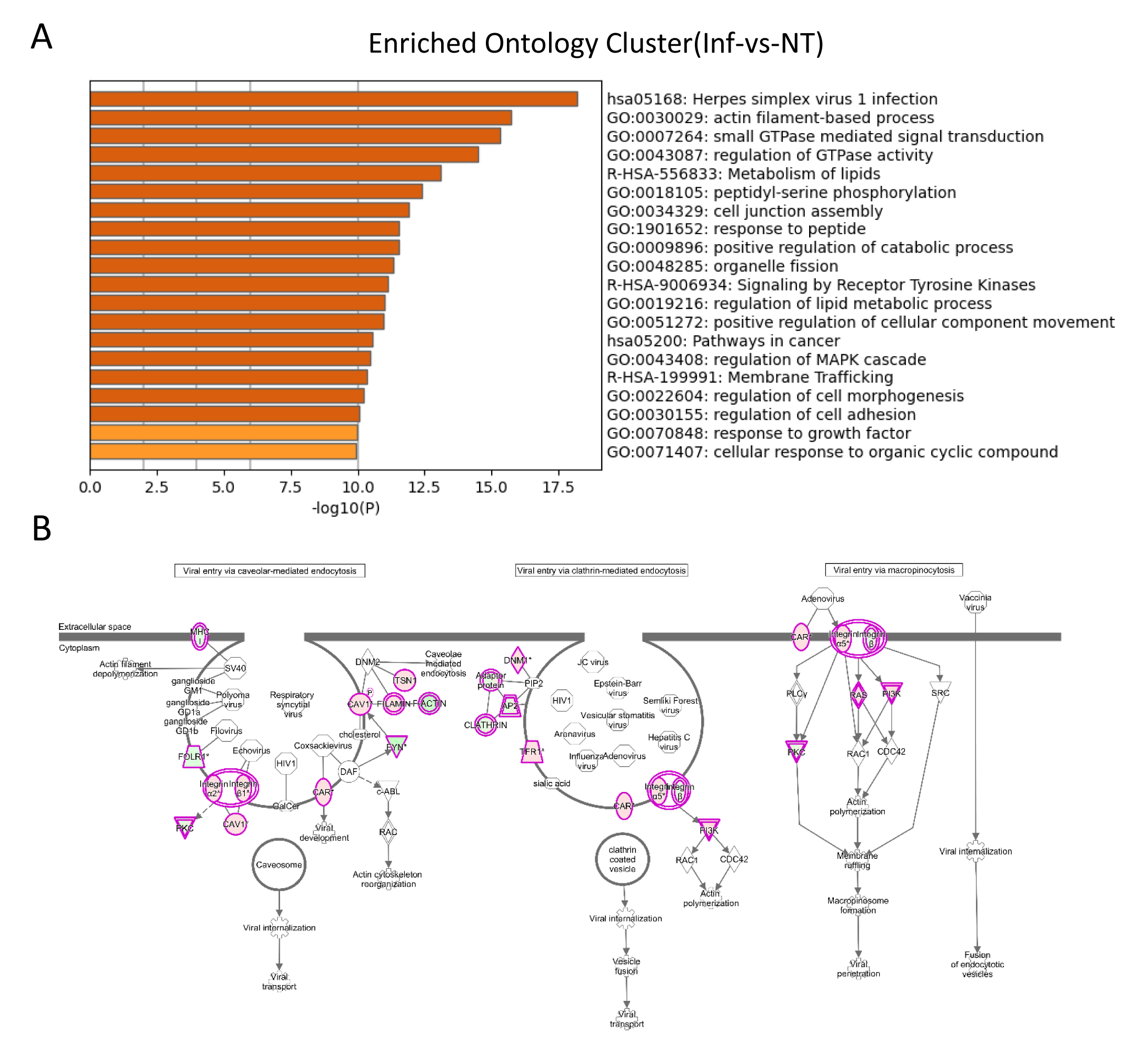

Supplement: Supplementary Figure 4 — Gene ontology and pathway patterns for host responses in HBECs following SARS-CoV-2 infection, compared with the control. (A) Top enriched ontology clusters following viral infection. (B) Viral entry related differentially expressed genes modulated in SARS-CoV-2 infected HBECs, compared with non-infected HBECs. [file Image_4.tif]

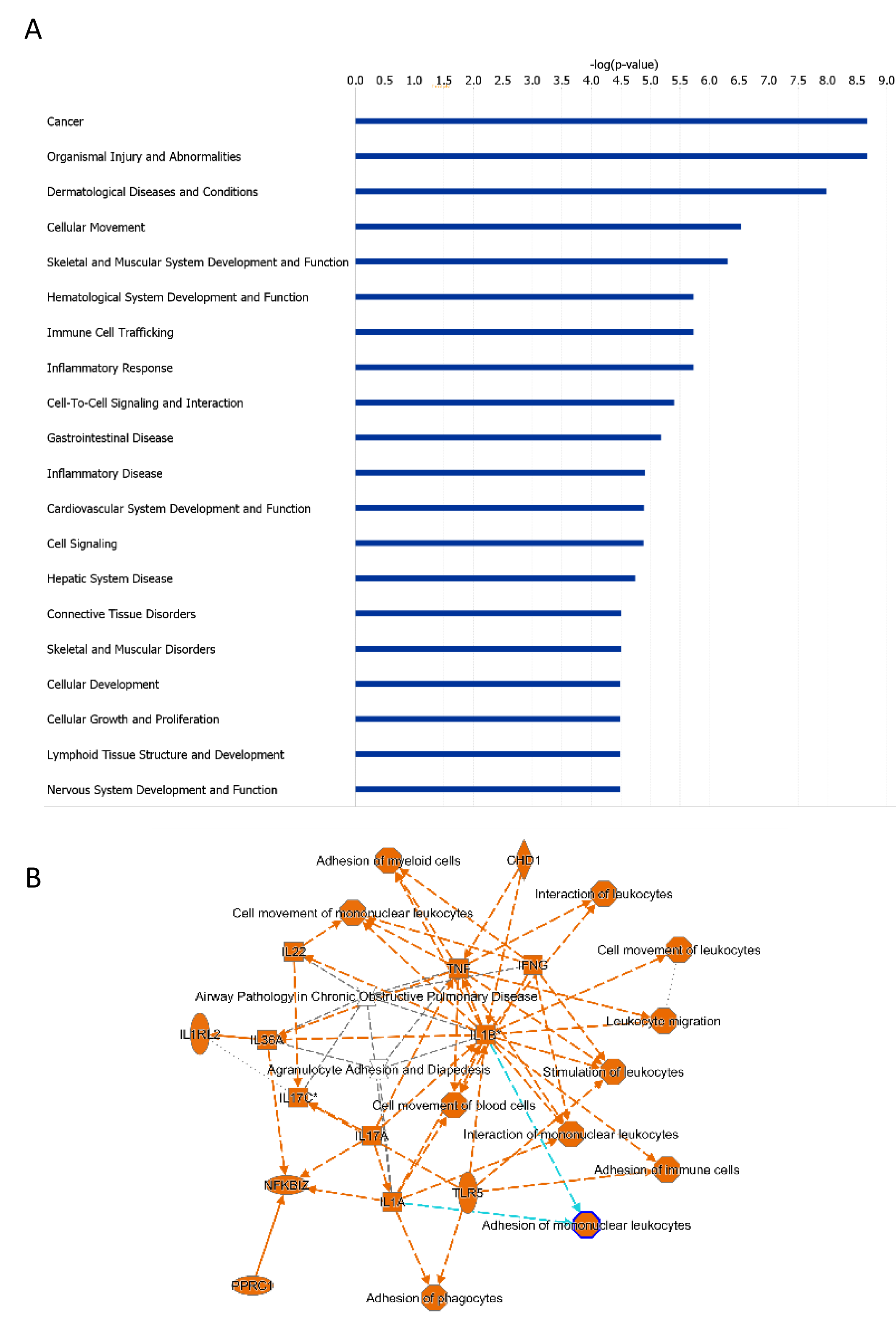

Supplement: Supplementary Figure 5 — Host responses in SARS-CoV-2–infected HBECs in the presence and absence of anti-RBD pAbs. (A) Top 20 enriched relevant diseases and functions modulated in HBECs in response to SARS-CoV-2 infection in the presence and absence of anti-RBD pAbs (p-value < 0.05); –log(P-values) are plotted on the x-axis. (B) Summary of enriched pathways following SARS-CoV-2 infection with or without anti-RBD pAb treatment. [file Image_5.tif]

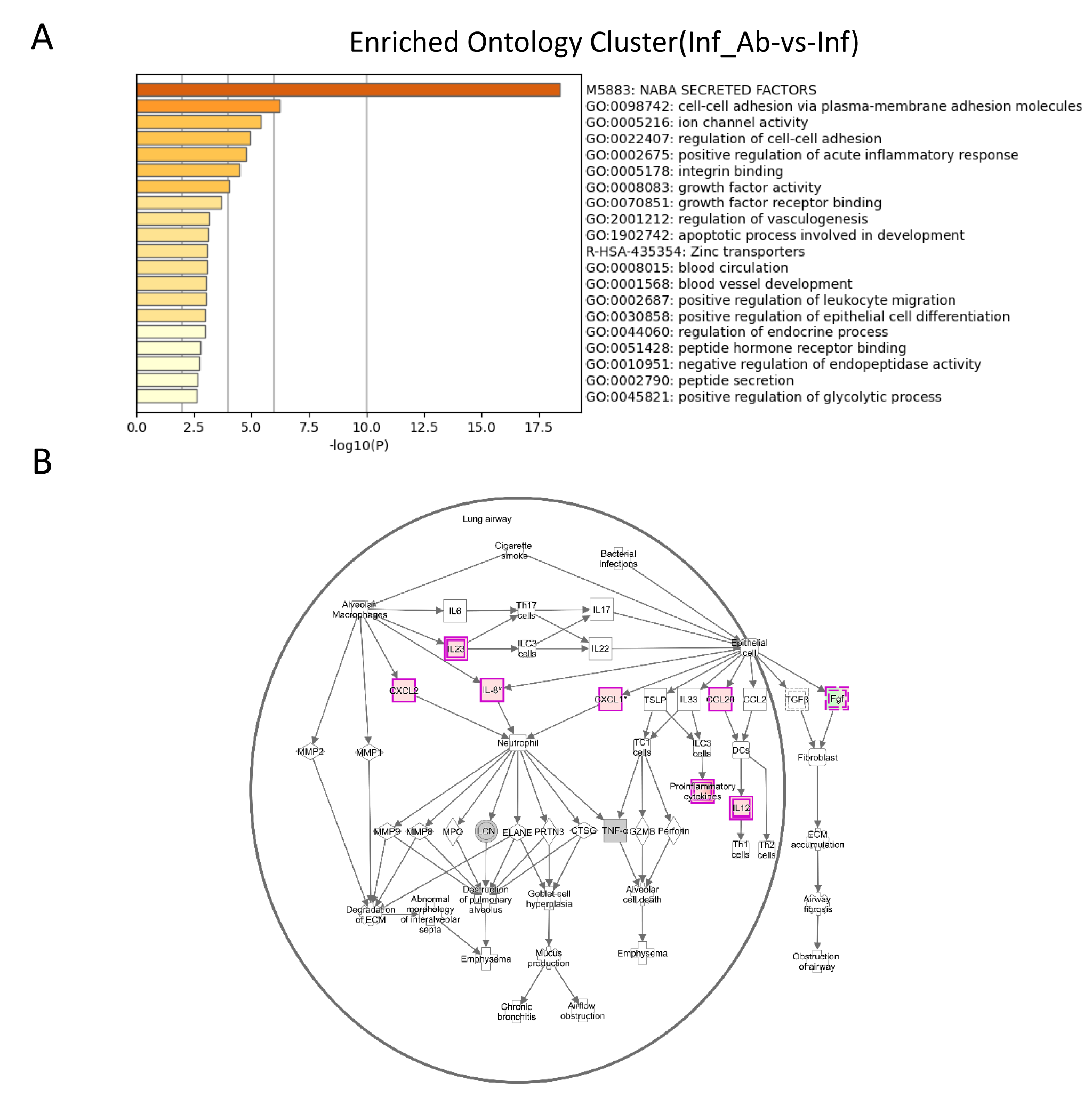

Supplement: Supplementary Figure 6 — Gene ontology and pathway patterns for host responses in SARS-CoV-2–infected HBECs in the presence and absence of anti-RBD pAbs. (A) Top enriched ontology clusters following SARS-CoV-2 infection in the presence and absence of anti-RBD pAbs. (B) Airway pathology–related differentially expressed genes modulated in SARS-CoV-2–infected HBECs combined with anti-RBD pAbs treatment. [file Image_6.tif]
